# Supplementary material for: Mechanical adaptability of artificial muscles from nanoscale molecular action
Source: Nat Commun. 2019 Oct 23;10:4819. doi: 10.1038/s41467-019-12786-2 (PMC6811622; doi:10.1038/s41467-019-12786-2)
Supplement: Supplementary file 1 — Supplementary Information [file 41467_2019_12786_MOESM1_ESM.pdf]

Mechanical adaptability of artificial muscles from nanoscale molecular action

Federico Lancia, Alexander Ryabchun, Anne-Déborah Nguindjel,
Supaporn Kwangmettatam, and Nathalie Katsonis*

Bio-inspired and Smart Materials, MESA+ Institute for Nanotechnology, University of Twente, PO Box 207, 7500 AE Enschede, The Netherlands

*Corresponding author: Nathalie Katsonis (n.h.katsonis@utwente.nl)

Supplementary Information

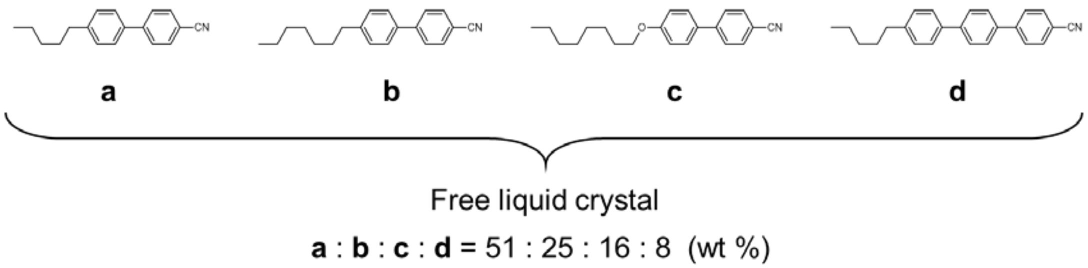

Supplementary Figure 1. Chemical composition of the free liquid crystal

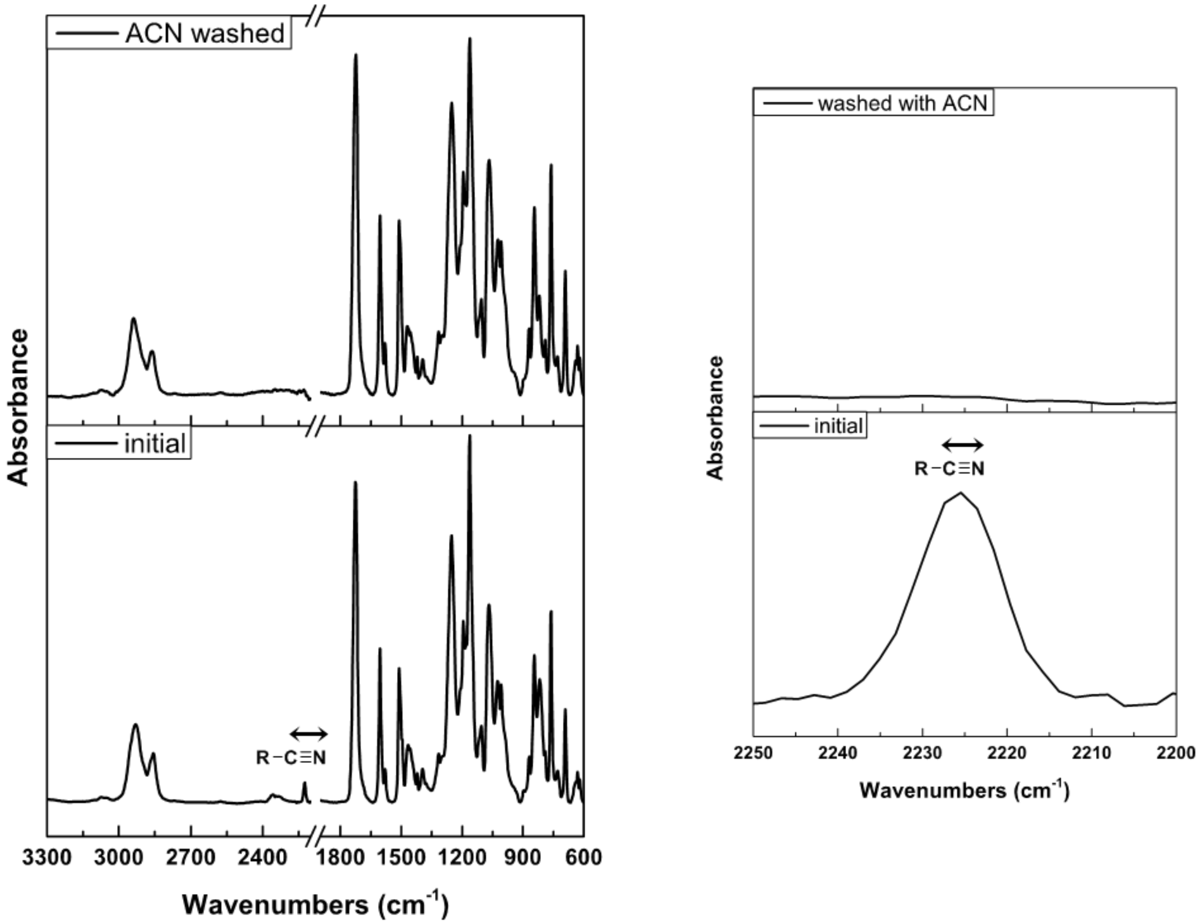

Supplementary Figure 2. ATR-IR spectra of film before (bottom) and after (top) washing with acetonitrile (ACN). On the right is a particular of the spectra on the absorption of nitrile.

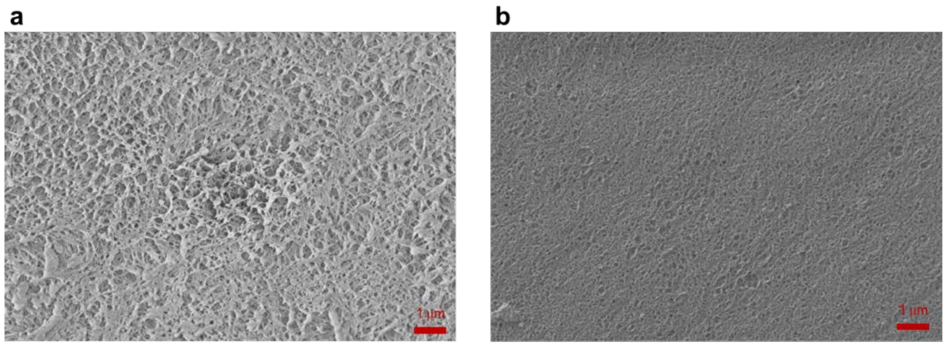

Supplementary Figure 3. Scanning electron microscopy images of cross section of liquid crystal film polymerized in the presence of **a**. 50 wt% and **b**. 25 wt% of free liquid crystal. The free liquid crystal was removed by washing the film with hexane. After drying the film was frozen in liquid nitrogen, and sliced.

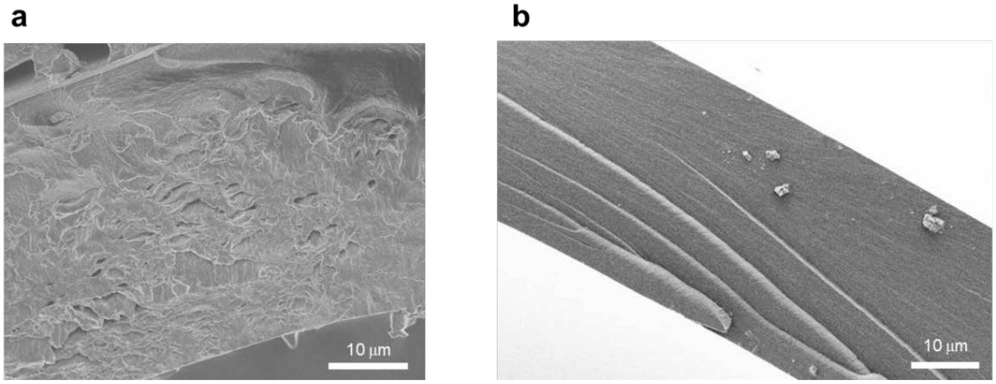

Supplementary Figure 4. Scanning electron micrographs. a. Cross section of a thin film of liquid crystal network polymerized in the presence of 8 wt% free liquid crystal. The free liquid crystal was removed by washing the film with hexane. After drying the film was frozen in liquid nitrogen, and sliced. **b**. Cross-section of a fully polymerized network.

Compound	R=1	R=1.9	R=2.9	R=3.8	R=4.7	R=5.6	R = 9.6
**C6BPN**	12.4 mol%<br>10.8 mg	12.3 mol%<br>10.8 mg	12.1 mol%<br>10.8 mg	12 mol%<br>10.8 mg	11.8 mol%<br>10.8 mg	11.7 mol%<br>10.8 mg	11.7 mol%<br>10.8 mg
**C6BP**	60.1 mol%<br>53 mg	56.6 mol%<br>50.5mg	53.1 mol%<br>48 mg	49.7 mol%<br>45.5 mg	46.4 mol%<br>43 mg	43.2 mol%<br>40.5 mg	32.3 mol%<br>30.3 mg
**C6M**	18.1 mol%<br>27.7 mg	17.8 mol%<br>27.7 mg	17.6 mol%<br>27.7 mg	17.4 mol%<br>27.7 mg	17.2 mol%<br>27.7 mg	17 mol%<br>27.7 mg	17 mol%<br>27.7 mg
**Azobenzene**	4.2 mol%<br>5mg	4.2 mol%<br>5mg	4.1 mol%<br>5mg	4 mol%<br>5mg	4 mol%<br>5mg	3.98 mol%<br>5mg	3.98 mol%<br>5mg
**E7**	4.1 mol%<br>2.5mg	8 mol%<br>5mg	12 mol%<br>7.5 mg	15.7 mg<br>10 mg	19.5 mol%<br>12.5 mg	23.1 mol%<br>15 mg	38.5 mol%<br>25 mg
**Irgacure 819**	1 mol%<br>1mg	1 mol%<br>1mg	1 mol%<br>1mg	1 mol%<br>1mg	1 mol%<br>1mg	1 mol%<br>1mg	1 mol%<br>1mg

Supplementary Table 1. Chemical composition of the polymerizable liquid crystals used in the study.

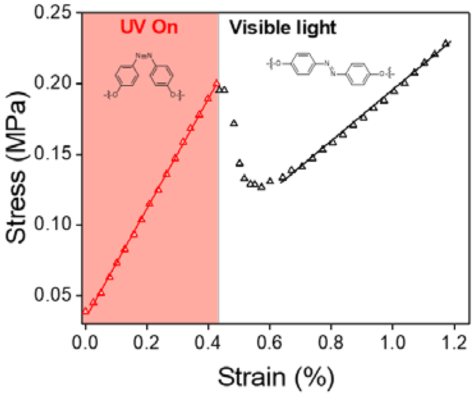

Supplementary Figure 5. Evidence for reversibility in photo-stiffening. In this experiment flat ribbons were used. The photostationary state was reached by illumination with λ = 365 nm over three minutes at 17 mW/cm^2. The reverse, *cis*-to-*trans* isomerization of the azobenzene is promoted by illumination with visible light (3 mW/cm^2), and leads to the material softening back.

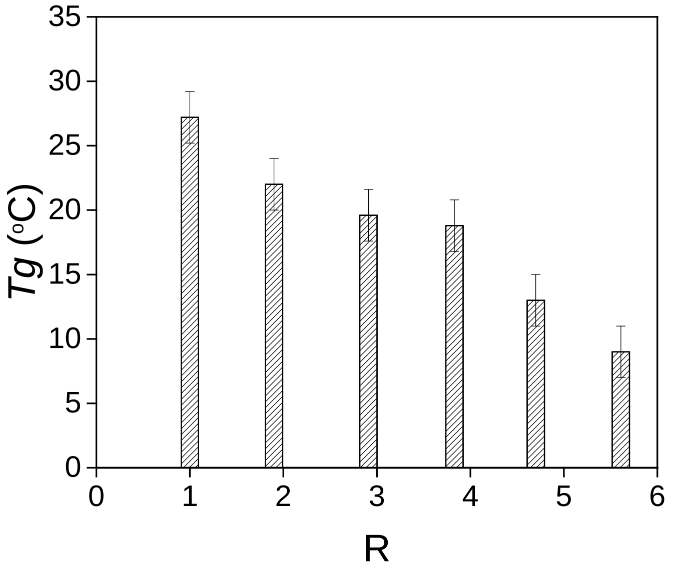

Supplementary Figure 6. Glass transition temperature (T_g) for mixtures with different *R*.

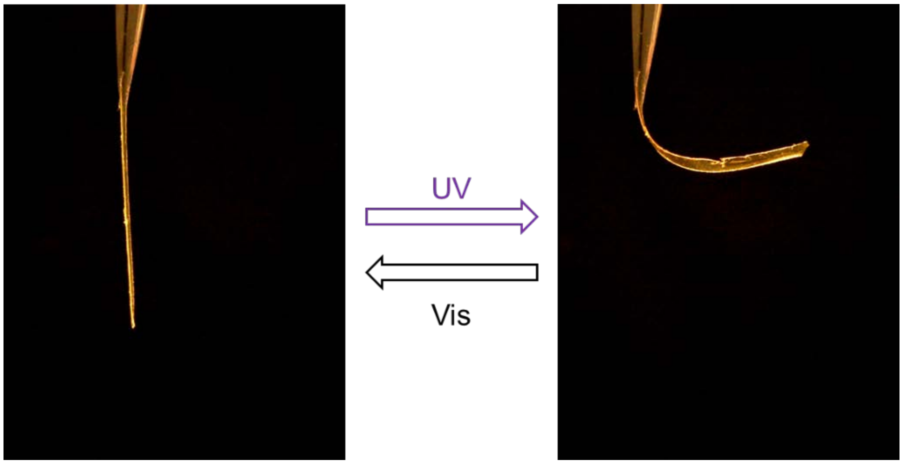

Supplementary Figure 7. Bending motion of a shape-shifting polymer upon illumination. The alignment of the liquid crystal is parallel to the long axis of the ribbon. The bending motion is mediated by a build-up in strain through the thickness of the polymeric ribbon.

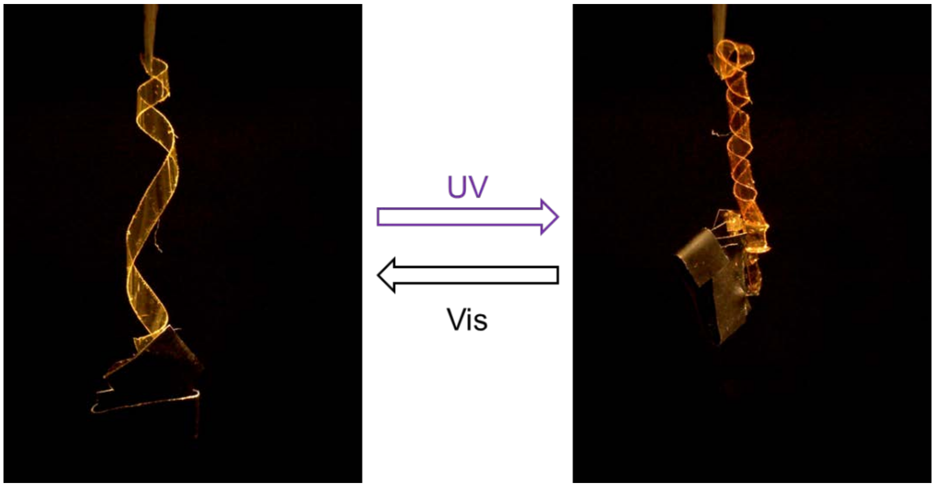

Supplementary Figure 8. Winding polymer spring lifting an object ca. 10.5 times its weight (11.67 mg mass of the object, 1.1 mg mass of the polymer spring). The work performed by the spring is ca. 0.64 μJ (6.1 J*mol^{-1} of Azo).

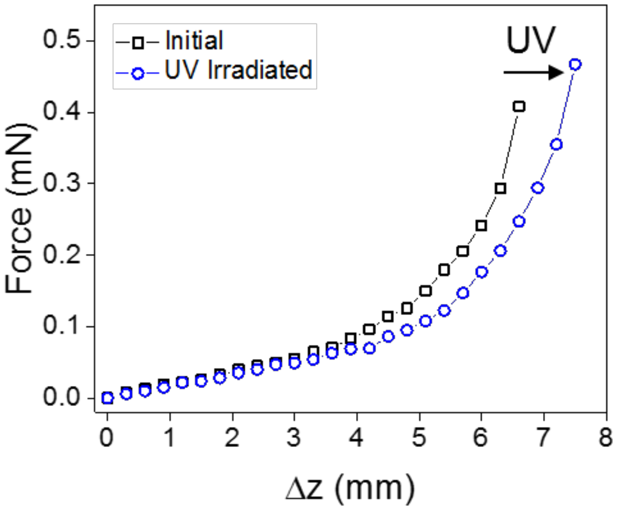

Supplementary Figure 9. Photoinduced softening of a liquid crystal polymer spring (R = 1). Nonlinear mechanical response (strain stiffening) of a liquid crystal polymer spring. The curve in black shows the force-displacement curve of the spring. The curve in blue shows the photo-induced softening upon UV irradiation.

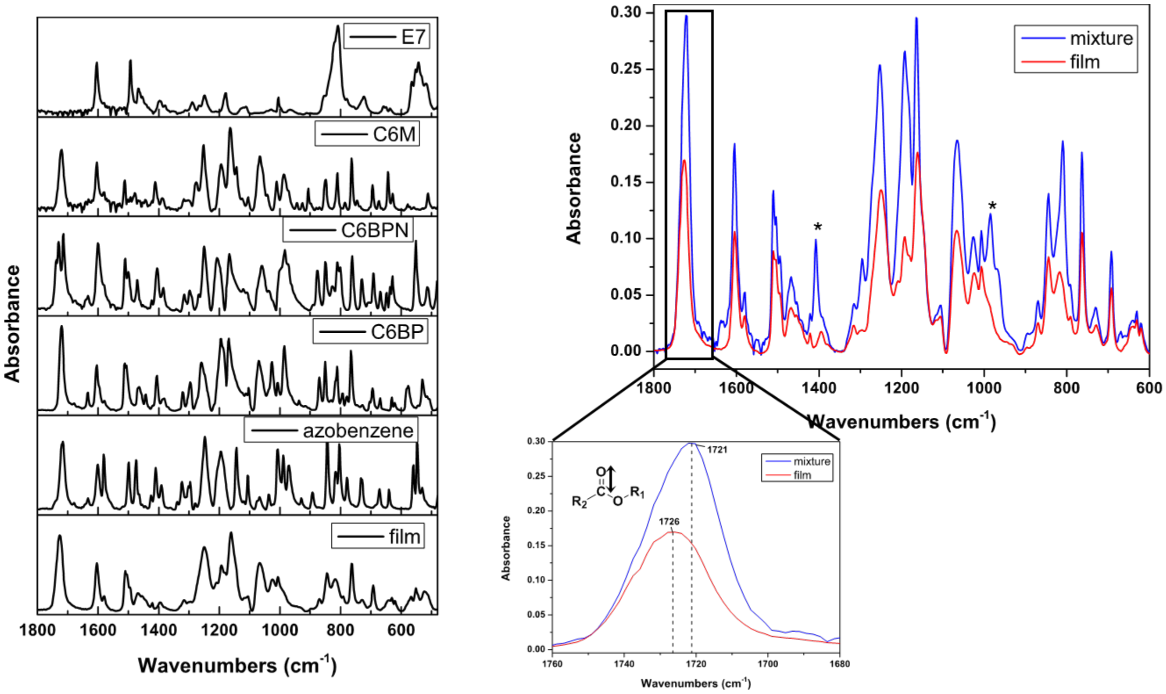

Supplementary Figure 10. (left panel) Infrared spectra for each component of the liquid crystal. (right panel) Infrared spectra of the liquid crystal mixture before (blue) and after polymerization (red).

Supplementary Method : Synthesis of the azobenzene diacrylate

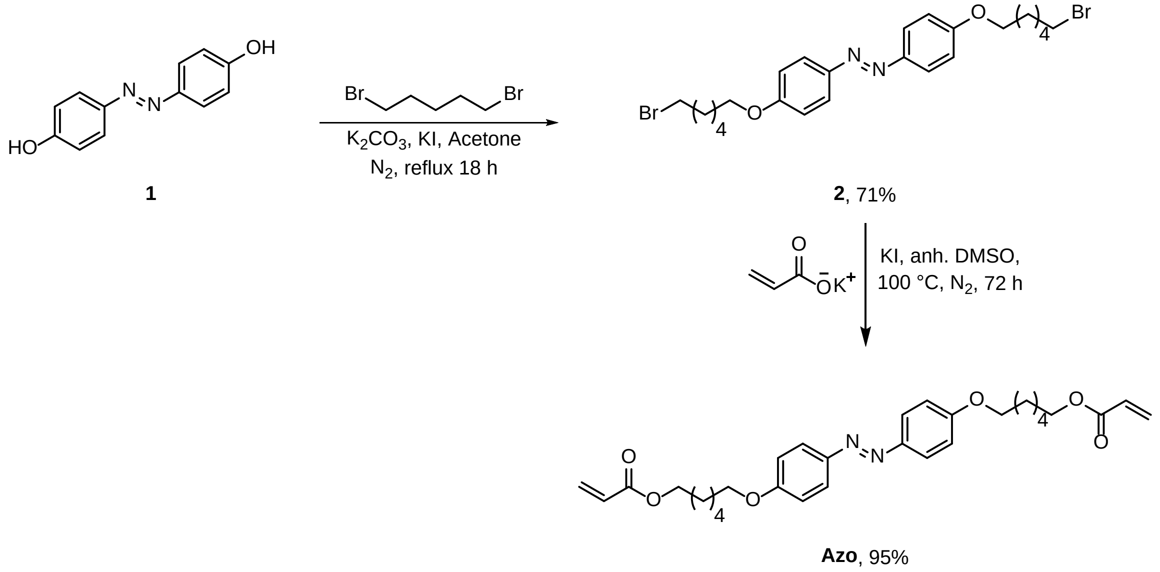

Compound 1. Compounds **1** was synthesized according previously reported procedure.[1]

Compound 2. Compound **2** was synthesized according previously reported procedure.[2]

Azobenzene diacrylate (Azo). Compound **2** (100 mg, 0.185 mmol), potassium acrylate (81.5 mg, 0.74 mmol), and potassium iodide (24.5 mg, 0.148 mmol) in anhydrous DMSO (5 mL) were stirred at 100 °C under N_2 atmosphere for 72 hours. After cooling down to room temperature, the reaction mixture was pour into water. The solution was filtered under vacuum and washed twice with water. The yellow solid was dissolved in dichloromethane/acetone and dried over Na_2SO_4. After filtration and evaporation of the organic solvent the azobenzene was isolated as a yellow solid (90 mg, 95%). ^{1}H-NMR (400 MHz, $CDCl_3$) δ 7.87 (d, J = 9.0 Hz, 2H), 6.98 (d, J = 9.0 Hz, 2H), 6.40 (dd, J = 17.3, 1.5 Hz, 1H), 6.12 (dd, J = 17.3, 10.4 Hz, 1H), 5.82 (dd, J = 10.4, 1.5 Hz, 1H), 4.18 (t, J = 6.6 Hz, 2H), 4.03 (t, J = 6.4 Hz, 2H), 1.92 – 1.78 (m, 2H), 1.77 – 1.67 (m, 2H), 1.58 – 1.41 (m, 4H).

---

[1] Bergen, A. *et al.* Photodependent melting of unmodified DNA using a photosensitive intercalator: A new and generic tool for photoreversible assembly of DNA nanostructures at constant temperature. *Nano Lett.*, **16**, 773–780 (2016).
[2] Zhang, Q. *et al.* New ionic liquid crystals based on azobenzene moiety with two symmetric imidazolium ion group substituents. *Liquid Crystals*, **35**, 299–1305 (2008).
